# Resident Memory-Like Tumor-Infiltrating Lymphocytes (TIL_RM_): Latest Players in the Immuno-Oncology Repertoire

**DOI:** 10.3389/fimmu.2018.01741

**Published:** 2018-07-26

**Authors:** Julian Smazynski, John R. Webb

**Affiliations:** ^1^Deeley Research Centre, BC Cancer Agency, Victoria, BC, Canada; ^2^Department of Biochemistry and Microbiology, University of Victoria, Victoria, BC, Canada

**Keywords:** resident memory T cells, CD8, CD103, tumor-infiltrating lymphocytes, prognosis

## Abstract

Resident memory T cells (T_RM_) are a recently identified subset of long-lived memory T cells that are characterized in terms of their unique surface phenotype combined with a non-recirculating pattern of localization to non-lymphoid, peripheral tissues. T_RM_ have quickly become a key area of focus in understanding immune responses to microbial infection in so-called “barrier” tissues, and appear to be particularly critical for protection against repeat exposure at the same site. More recently, tumor-infiltrating T cells with canonical T_RM_ features are being identified in human cancers, in particular cancers of epithelial origin, and their presence is broadly found to be associated with favorable long-term prognosis. Moreover, recent studies have shown that these “resident memory-like” tumor-infiltrating lymphocytes (referred to herein as TIL_RM_) are uniquely activated in melanoma patients undergoing PD-1 directed checkpoint blockade therapy. Accordingly, there is much interest at present regarding the biology of these cells and their precise role in anti-cancer immunity. Herein, we review the current state of the literature regarding TIL_RM_ with a specific emphasis on their specificity, origins, and relationship to conventional pathogen-specific T_RM_ and speculate upon the way(s) in which they might contribute to improved prognosis for cancer patients. We discuss the growing body of evidence that suggests TIL_RM_ may represent a population of bona-fide tumor-reactive T cells and the attractive possibility of leveraging this cell population for future immunotherapy.

## Brief Introduction to Resident Memory T Cells (T_RM_) and the T_RM_-Defining Surface Marker CD103

In recent years, there has been growing recognition of the importance of a peripheral, non-recirculating component of the immune system known as T_RM_ [for review see Ref. (1–3)]. T_RM_ have historically been defined by their peripheral tissue localization and lack of circulatory activity. More recently, there is increasing understanding of the unique surface phenotype(s) of T_RM_ and how the specific molecules that comprise this phenotype contribute to their (non-)circulatory nature. Although this phenotype can vary somewhat between tissues, disease states, and CD4 versus CD8 subsets, most T_RM_ in skin, lung, and GI tract typically express CD69, a molecule widely considered to be an indicator of recent activation, but which is also involved in downregulation of the receptor for sphingosine 1 phosphate (S1P1), thereby inhibiting the ability of T_RM_ to traffic out from peripheral tissue in response to S1P1 gradients (4). Likewise, T_RM_ frequently lack surface expression of CCR7, preventing them from trafficking in response to gradients of CCL19 and CCL21 (5). In addition, surface expression of CD103 (the α_E_ component of the α_E_/β_7_ integrin molecule) (6, 7) is now widely considered to be a canonical marker of T_RM_, and although T_RM_ populations can be comprised of variable proportions of both CD4 and CD8 cells, CD103 appears to be uniquely overexpressed by CD8 T_RM_ (8). CD103 expression is also biologically relevant to the non-recirculating phenotype of T_RM_, as the ligand for α_E_(CD103)/β_7_ integrin is E-cadherin expressed on epithelial cells (9). Although chemokines are thought to be the initial mediator of T cell recruitment into peripheral sites of inflammation, adhesive interactions between α_E_(CD103)/β_7_ and E-cadherin is thought to be responsible for the long-term “retention” of antigen-specific T_RM_ at relevant sites (10, 11). This phenomenon is particularly well studied in the context of mucosal tissue infection, where the long-term retention of T_RM_ at the site of an initial infection is thought to provide durable and rapid protection against repeat attack by the same organism. Indeed, once T_RM_ populations are established, they can be retained at the original site of infection for months or even years, even in the complete absence of relevant antigen (12–14). This T_RM_ phenomenon can also be exploited by vaccination strategies that involve delivery of vaccine to the relevant mucosal tissue (15). Indeed, the historical field of “mucosal” immunity and the newer field of “T_RM_-mediated” immunity are rapidly merging in terms of the memory T cell components.

In addition to mediating adhesion and T_RM_ formation, both α_E_(CD103)/β_7_ and E-cadherin are also capable of intracellular signaling. For example, the intracellular domain of E-cadherin interacts with β-catenin which in turn interacts with the actin cytoskeleton, affecting cell shape and motility (16). Likewise, cross-linking of surface-expressed α_E_(CD103)/β_7_ impacts the shape and motility of lymphocytes (17), enhances T cell proliferation and induces lysis of target cells (18). Thus through the combination of “inside-out” and “outside-in” signals, α_E_(CD103)/β_7_ has the potential to profoundly impact T_RM_ effector function, in addition to augmenting peripheral memory formation.

## Mechanism of CD103 Upregulation on T_RM_

TGF-β has long been known to play a key role in the regulation of α_E_(CD103)/β_7_ surface expression on T lymphocytes (19, 20). Although TGF-β is often considered solely as an immunosuppressive factor, it is, in reality, a highly pleiotropic cytokine that is expressed in a multitude of (primarily peripheral) tissue types and has biological activities that are context specific (21). Interestingly, although TGF-β is required for upregulation of α_E_(CD103)/β_7_ surface expression, TGF-β exposure alone is not sufficient (18, 22). Rather, it is the combination of TGF-β plus concurrent signaling through the TCR that results in dramatic and rapid α_E_(CD103)/β_7_ expression. Indeed, the combination of these two signals makes perfect sense biologically as it would allow for large numbers of lymphocytes (with diverse specificities) to transiently traffic through TGF-β-rich sites of peripheral infection, but result in the α_E_(CD103)/β_7_-mediated retention of only those T cells with relevant specificity. This model of T_RM_ formation is supported by the finding that in CD103 knockout mice, numbers of T_RM_ are substantially reduced (10). Likewise, dysregulation of the SMAD signaling pathway downstream of the TGF-β-receptor results in reduced numbers of T_RM_ (23).

Although TGF-β-mediated upregulation of CD103 clearly plays an important role in the establishment of T_RM_, it is certainly not the only mechanism. For example, it has also been reported that the formation of T_RM_ populations can be enhanced through signaling *via* the homeostatic cytokine, interleukin-15 (IL-15) (24, 25). However, dependency upon IL-15 for T_RM_ formation varies from tissue to tissue (26), implying that the requirement for IL-15 is not absolute and may be more complex than that of TGF-β. Moreover, as described above, CD4^+^ T_RM_ populations, in general, express much lower levels of CD103 than do CD8^+^ T_RM_, thus they must maintain residency in a CD103-independent manner (27, 28).

## T_RM_ in the Cancer Setting

In recent years, there has been growing appreciation that T_RM_ biology/immunology is not unique to the infectious disease setting. Indeed, it has long been speculated that T_RM_ play a key role in both allograft rejection and autoimmunity. For example, α_E_(CD103)/β_7_ is expressed on the majority of tissue-infiltrating CD8^+^ T cells during transplant rejection (20, 29, 30) and graft versus host disease (22). In CD103-deficient mice, T cells are not able to infiltrate allogeneic islet cell transplants and allografts persist for long periods *in vivo* (30, 31) often surrounded by a characteristic “halo” of CD103-deficient CD8 T cells. In the autoimmune disease setting, islet infiltrating cells in both human diabetic patients (32, 33) and mouse models of autoimmune diabetes (34) are enriched for α_E_(CD103)/β_7_-expressing T_RM_. Presumably, in each of these settings T_RM_ are derived *via* the same TGF-β plus concurrent TCR signaling mechanism described above for infectious diseases.

α_E_(CD103)/β_7_-expressing tumor-infiltrating T cells (TIL) are also now turning up, with increasing regularity, in various cancer settings, particularly in cancers of epithelial origin. This should really not be surprising considering the relationship between TGF-β and α_E_(CD103)/β_7_ and the frequent expression of TGF-β in cancers of various types. TGF-β overexpression in cancer has been broadly considered as an immunosuppressive mechanism of tumor escape from immunological pressure (21, 35). However, an alternate hypothesis could be that TGF-β production by tumors is not so much an acquired trait as it is an amplification of the TGF-β that is expressed as part of the “normal” biology of epithelial tissues. Regardless of the mechanism, when tumor-reactive T cells enter these TGF-β-rich environments and then become activated through the TCR, there is full reason to assume they would upregulate α_E_(CD103)/β_7_ on the cell surface, in the same manner that conventional T_RM_ do.

However, as described above, CD103 expression is only one part the larger phenotypic profile that defines T_RM_. Whether CD103-expressing TIL are phenotypically identical to conventional pathogen-specific T_RM_, or whether they are simply closely related cousins is an issue that remains to be determined. For example, the phenotypic features that are known to be shared among conventional T_RM_ populations, regardless of their specificity and/or tissue location, are reported to be driven by the T_RM_ master transcriptional regulators Blimp-1 and Hobit (36). However, the expression of Blimp-1 and Hobit in tumor-infiltrating T_RM_ is yet to be reported. By contrast, the transcription factor Runx3, which influences the downregulation of mRNA transcripts associated with cellular migration (S1pr1, Klf2, and Ccr7) appears to be expressed in both conventional and tumor-infiltrating T_RM_ (37). Moreover, conventional pathogen-specific T_RM_ are thought to be retained in peripheral tissue after resolution of infection, acting as a vanguard against future re-exposure. In this context, a large proportion of conventional T_RM_ are likely persisting in peripheral tissue in an antigen-free manner, until such time as they become re-challenged through re-exposure. By contrast, tumor-infiltrating T_RM_ (assuming they are tumor-specific) are resident within active tumor tissue and would thus be continuously exposed to antigen, which would likely result in a phenotype distinct from conventional “resting” T_RM_. For these reasons and because the precise relationship between conventional T_RM_ and tumor-infiltrating T_RM_ is yet to be well-defined in the literature, in our laboratory we have adopted the term “TIL_RM_” (resident memory-like TIL) to delineate these CD103-expressing tumor resident cells from conventional pathogen-specific T_RM_.

Until recently, broader investigation into the global nature of TIL_RM_ infiltration in human tumors was severely hampered by the lack of an anti-human CD103 antibody that was suitable for IHC of formalin-fixed tissues. This situation changed in 2013 when a new antibody was, ironically, developed for diagnosis of hairy cell leukemia (38), a setting where CD103 is ectopically overexpressed. Since the introduction of this reagent, TIL_RM_ have now been reported to be present in at least eight different tumor settings including lung, breast, ovarian, endometrial, cervical, melanoma, colorectal, pancreatic, and bladder cancer (39–53) (see Table 1). In the majority of these reports, CD103 is used as a marker to delineate “intraepithelial” TIL, and more importantly, the presence of CD103^+^ TIL is associated with favorable prognosis.

**Table 1:** Summary of studies examining CD103^+^ TIL_RM_ as a prognostic indicator in solid cancers.

Tumor histology	Summary	Reference
Bladder	A large proportion of TIL in the urothelium co-express CD8^+^ CD103^+^. Carcinoma stromal tissue was highly enriched for CD8^+^ CD103^+^ TIL but not associated with increased E-cadherin expression	Cresswell et al. (50)

Colorectal	Microsatellite instable tumors show increased infiltration of CD8^+^ CD103^+^ TIL compared to microsatellite stable tumors	Quinn et al. (47)

Colon	CD103 expression is enhanced by antigen recognition and TGF-β signaling. T cell activation in the presence of TGF-β induces CD103 expression	Ling et al. (49)

Ovarian	CD103^+^ TIL were found to be abundant across all major ovarian cancer subtypes but highly enriched in high-grade serous cancer (HGSC), and their presence correlates with improved survival	Webb et al. (55)

Lung	CD103^+^ TIL correlate with improved early stage patient survival in non-small cell lung cancer (NSCLC) and intraepithelial TIL density. CD103^+^ TIL show enhanced effector function against autologous tumor	Djenidi et al. (39)

Ovarian	CD103 demarcates intraepithelial CD8^+^ TIL which co-express PD-1 and appear quiescent in the tumor microenvironment	Webb et al. (41)

Breast	High abundance of CD103^+^ TIL in ER negative (basal-like subtype) tumors within intraepithelial regions correlates with good prognosis	Wang et al. (40)

Melanoma	Interlesional TIL populations show an enriched gene signature indicative of a resident memory phenotype which is responsive to immune checkpoint blockade	Boddupalli et al. (48)

Endometrial	Abundance of CD8^+^ CD103^+^ TIL in endometrial tumor epithelium is a strong prognostic indicator in endometrial adenocarcnoma	Workel et al. (42)

Ovarian	CD103^+^ TIL collected from HGSC co-express PD-1 and CD27. TIL activated in the presence of HGSC upregulate CD103	Komdeur et al. (43)

NSCLC and head and neck squamous cell cancer	Cytotoxic T lymphocytes have an enriched resident memory gene signature. CD8^+^ CD103^+^ TIL co-express checkpoint receptors such as PD-1 and CTLA-4. Higher density of resident memory T cells (T_RM_)-like TIL are associated with improved patient outcome	Ganesan et al. (46)

Cervical	CD103 gene expression is associated with effector T cell function. Abundance of intraepithelial CD8^+^ CD103^+^ TIL correlates with improved patient survival	Komdeur et al. (44)

Pancreatic	Increased ratio of CD8^+^ CD103^+^ TIL to CD8^+^ CD103^−^ TIL correlates with improved patient survival	Lohneis et al. (51)

Melanoma	Presence of CD8^+^ CD69^+^ CD103^+^ TIL correlates with improved patient survival in melanoma. CD103^+^ TIL show high levels of expression of the inhibitory markers PD-1 and LAG-3	Edwards et al. (45)

Lung	Single-cell RNA sequencing of lung TIL showed distinct pre-exhausted and exhausted TIL phenotypes. Tumor resident T cells expressed high levels of CD69 and CD103 overall	Guo et al. (52)

Breast	Single-cell RNA sequencing of breast TIL revealed high TIL abundance was characterized by a T_RM_-like phenotype and associated with improved patient survival in triple negative breast cancer	Savas et al. (53)

## TIL_RM_ Cells in the Gynecologic Cancer Setting

Our group first noted the presence of TIL_RM_ cells in the ovarian cancer (OvCa) setting during a flow cytometry-based survey of immune cells present in OvCa patient ascites (54). Interestingly, some but not all, ascites specimens contained CD103-expressing T cells, specifically within the CD8 subset and sometimes comprising as much as 80% of the cells in that compartment. The presence of these cells in a fluid-based tissue (ascites) initially seemed inconsistent with them being a T_RM_ population as T_RM_ are normally restricted to solid tissues. However, the ascites compartment in ovarian patients can contain large numbers of free-floating tumor cells plus abundant amounts of TGF-β. Thus it should not be surprising that tumor-specific T cells present in this fluid compartment could adopt a T_RM_ phenotype more typical of solid tissues. We have also found that these cells have a unique phenotype that includes upregulation of HLA-DR, Ki67, and PD-1, but a lack of CD69, CD137, or intracellular cytokines suggesting that they have been recently activated, but are not actively “engaging” with targets at the time of analysis. Although the cells were PD-1 positive (41), they lacked other markers of exhaustion and were capable of robust cytokine production after stimulation with PMA/ionomycin, *ex vivo*, suggesting that they were not terminally exhausted. These initial findings regarding CD103-expressing TIL_RM_ in OvCa were limited to flow cytometric analysis of small numbers of ascites specimens. However, once an IHC-suitable antibody was available, we followed up by analyzing larger cohorts of patients using tissue microarray technology and showed that CD103-expressing TIL_RM_ cells were also present in the solid tumors of some, but not all, OvCa patients (55). Moreover, we also demonstrated that infiltration of tumors by TIL_RM_ correlated strongly with a favorable 5-year disease-specific survival advantage in high-grade serous cancer (HGSC), the most lethal of OvCas (55, 56). This finding has now been replicated in three additional cohorts of OvCa patients (43, 57, 58) as well as in endometrial (42) and cervical cancers (44). Clearly, TIL_RM_ cells are playing an important role in the gynecologic tumor setting, as they are in other epithelial tumor settings.

## Evidence in Support of TIL_RM_ Cells Being “Tumor-Specific”

Based upon their significant prognostic benefit and unique surface phenotype, we and others speculate that TIL_RM_ in OvCa as well as other cancers are highly likely to be tumor-specific (56). Unfortunately, at present there is a paucity of well-characterized tumor antigens in the HGSC setting to directly test this hypothesis. Nonetheless, our group has previously characterized the cellular immune response to the cancer/testis tumor antigen (NY-ESO-1) in a small cohort of HGSC patients (59) by IFN-γ ELISPOT. The specificity of one such patient was mapped to a well-known HLA-A2-restricted epitope (NY-ESO-1_157–165_) for which MHC tetramer reagents are available. Combining tetramer staining with CD103 staining revealed that NY-ESO-1-specific CD8^+^ cells in this tumor sample were indeed CD103^+^ (54), confirming that tumor-specific cells fell within the TIL_RM_ compartment in this patient. However, the NY-ESO-1-specific cells in this sample comprised only a tiny proportion of the entire TIL_RM_ population, which had otherwise unknown specificity.

Similar results regarding tumor specificity of TIL_RM_ have been obtained in other cancer settings. One of the first studies to demonstrate tumor specificity of TIL_RM_ was in the non-small cell lung cancer setting wherein the authors found that CD8^+^CD103^+^ TIL selectively upregulated CD107a and granzyme B in the presence of autologous tumor cells and also specifically lysed autologous tumor cells when co-cultured in the presence of an anti-PD-1 blocking antibody (39). More recently, TIL_RM_ populations have been identified in the melanoma setting and have been shown to contain cells that stain with melan A-specific tetramers (45), again confirming the presence of tumor-specific T cells in the TIL_RM_ subset. Likewise, TIL_RM_ have been demonstrated to play a role in anti-tumor immunity in various murine tumor models. For example, using a murine model of melanoma it was reported that CD103 was required for establishment of gp100-specific TIL_RM_ populations at the tumor site (60). Interestingly, in this model gp100-specific TIL_RM_ cells even remained at the site after tumor resolution and provided long-term immunity against rechallenge, but also caused permanent vitiligo in the dermis. On a somewhat related note CD103^+^ TRM have also recently been reported to be abundant in human vitiligo specimens (61).

Despite the abundance of evidence supporting the likely tumor specificity of TIL_RM_, one should also consider the alternate hypothesis, that because many of these epithelial tumor types originate from a tissue that could be directly or indirectly considered a mucosal barrier tissue, the TIL_RM_ populations could actually be conventional pathogen-specific T_RM_ “bystander” populations that have been amplified during tumor outgrowth. Indeed, this possibility has been raised in a very recent study designed to characterize the phenotypes of authentic tumor-specific TIL versus bystander virus-specific TIL present in human colorectal and lung tumors (62). Interestingly, in this study both the tumor-specific and bystander T cells were found to express features of T_RM_, including CD103, whereas CD39 was found to be a more reliable marker for distinguishing between the two. Although this study does not contradict earlier findings demonstrating CD103 expression by tumor-reactive TIL, if correct, it suggests that TIL_RM_ populations may actually be more heterogeneous than previously thought. Indeed this might be particularly relevant in the gynecologic cancer setting as HSV-2 reactive T cells with a typical T_RM_ phenotype have been reported to be present in the cervical tissue of women with known HSV-2 infection (63) and the numbers of typical T_RM_ in the fallopian tube are reported to increase with age (64). Perhaps these pathogen-specific TIL_RM_ populations in previously healthy gynecologic barrier tissues simply “come along for the ride” once the tissue becomes cancerous, and perhaps even co-exist with nascent tumor-specific TIL_RM_ populations. Clearly, it remains a challenge to the field to more precisely define the specificity of TIL_RM_.

## The “Paradox” of the Prognostic Effect of TIL_RM_

As described above, the significant prognostic benefit conferred by TIL_RM_ in HGSC and other cancers implies that they are likely to be tumor-specific or at least encompass tumor-specific populations. However, at the same time, this interpretation is somewhat paradoxical as these cells are present in tumor specimens that have been obtained from patients who have required clinical intervention (in the form of surgical de-bulking in the case of HGSC). This scenario suggests that if TIL_RM_ are indeed tumor-specific, they have ultimately lost the ability to control growth of the primary tumor. In recent years, it has become readily apparent that this paradox can be explained, at least in part, by various mechanisms of immune suppression and/or immune exhaustion. Indeed, the tumor microenvironment in OvCa, much like other cancers, has long been considered to be highly immunosuppressive due to the presence of soluble immune-inhibitory factors including IL-10, TGF-β, IDO, and PGE-2 (65). Likewise, the master immune-inhibitory switch molecule CTLA-4 has also been shown to be upregulated in the OvCa setting (66). In addition, inhibitory cells such as CD4^+^ Foxp3^+^ regulatory T cells (67), immunosuppressive B7-H4^+^ tumor-associated macrophages (68), and myeloid-derived suppressor cells (69) have all been reported to be present in OvCa. More recently, the PD-1 immune checkpoint pathway has also been found to play a potential role in OvCa (70), as it has in many other cancer settings.

**Figure 1:**
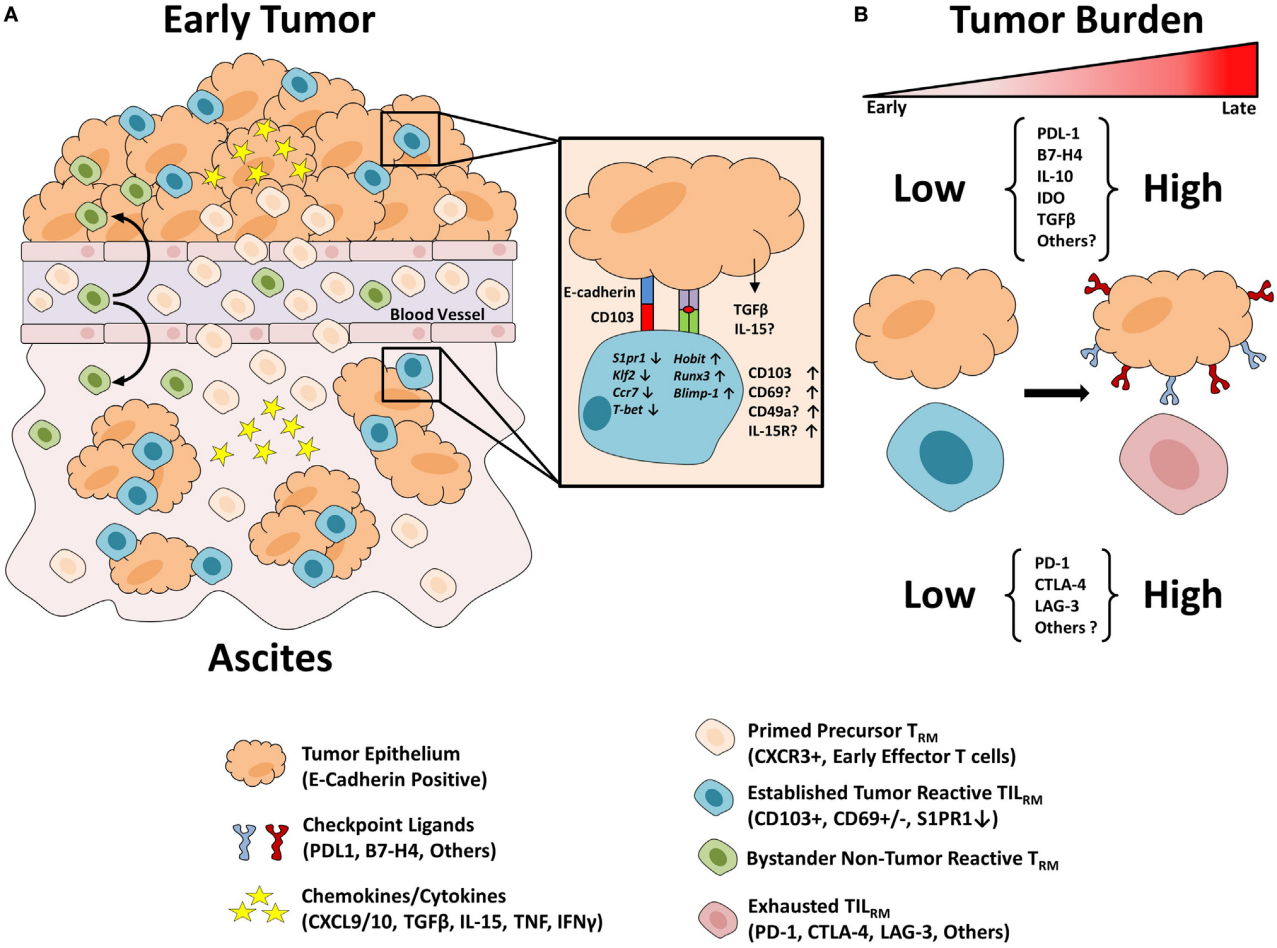
Proposed model of TIL_RM_ formation. **(A)** Precursor resident memory T cells (T_RM_) populations are composed of previously activated CXCR3^+^ T cells which are attracted to the chemokines CXCL9/10 in the inflamed tumor environment. Within the epithelial tumor tissue, cells encounter TGF-β which promotes CD103 expression. In response to TCR engagement cells may express increased CD69 which in turn disrupts S1PR1 expression leading to a breakdown in the chemoattractant signal from S1P concentrations in the blood. TIL_RM_ cells bind to their target tumor cells with increased strength due to CD103 binding to its ligand E-cadherin, thus promoting their residency in the epithelial tissue. Similarly, precursor T_RM_ may traffic to the inflamed ascites environment and interact with epithelial tumor cells leading to TIL_RM_ formation. Finally, bystander precursor T_RM_ populations may traffic to the inflamed tumor and/or ascites environment and develop T_RM_-like characteristics but with irrelevant antigen specificity. **(B)** Throughout cancer progression, the tumor microenvironment becomes increasingly inhospitable with increased tumor burden. Tumor cells upregulate immunosuppressive checkpoint receptors to avoid immune eradication. Following T cell activation and prolonged antigen stimulation T cells upregulate a variety of immune checkpoints which act to suppress anti-tumor immunity. TIL_RM_ may be inhibited due to the high expression of such checkpoint receptors and thus are likely candidates to respond to immune checkpoint blockade therapy.

As mentioned above, our group has recently made the observation that the CD103^+^ TIL_RM_ in HGSC tumors (and ascites) are almost universally positive for PD-1 surface expression (41). By contrast, PD-1 surface expression does not seem to be a universal characteristic of conventional T_RM_ where expression of PD-1 is reported to be dynamic and perhaps even restricted to certain tissue types (71, 72). This finding would suggest that unlike conventional T_RM_, intra-tumoral TIL_RM_ may have become partially (or permanently) exhausted likely due to chronic stimulation with tumor antigen over a period of weeks to months. Indeed, we speculate that although CD103 expression may initially be beneficial to TIL_RM_ function by promoting retention within the tumor, CD103 may actually be detrimental in the longer term by causing T cells to become “trapped” within the tumor, thereby exacerbating the phenomenon of chronic Ag stimulation (see Figure 1). This scenario is supported by a recent finding in melanoma, wherein CD103^+^ TIL_RM_ selectively and specifically became activated and started expanding in patients who were undergoing anti-PD-1 immunotherapy (45). This finding suggests that TIL_RM_ may be critical players in dictating responsiveness to checkpoint blockade therapy, a topic which is currently undergoing intense scrutiny. Thus, more fully understanding the biology of TIL_RM_ becomes paramount in that context.

## Conclusion and Future Perspectives

Resident memory T cells have rapidly gained a reputation as sentinels of peripheral immunity, primed to prevent infection *via* re-exposure to a previously encountered pathogen. However, the biology of T_RM_ is now spilling over into the field of oncology where T_RM_ are being detected in an increasing number of tumor settings. Whether all the functions and characteristics of conventional T_RM_ directly translate into the unique, dynamic and often hostile microenvironment of tumors has yet to be fully elucidated. Furthermore, what role TIL_RM_ play in preventing disease recurrence after standard treatments such as radiation and chemotherapy is essentially unknown territory. Clearly much remains to be learned about these cells. What is certain is the prognostic benefit that comes along with the presence of TIL_RM_, implying that at best, they play a direct role in anti-tumor immunity, or at minimum, they are a surrogate indicator of a separate phenomenon that leads to favorable outcomes for patients with TIL_RM_ positive tumors. Future studies should explore the potential utility of these cells in cancer immunotherapy strategies, including checkpoint blockade, cancer vaccination, and cellular therapies. Of particular interest would be understanding methodologies to convert immunologically “cold” tumors to “warm” ones by coaxing the formation and putative anti-tumor activity of TIL_RM_. One can even imagine that the TIL_RM_ phenomenon could be applied to the rapid growing field of chimeric antigen receptor (CAR) T cell technology as it transitions into the solid tumor setting, by facilitating the retention of CAR T cells in solid tumor targets. Clearly, we are still in the early days of understanding TIL_RM_ biology, but the potential implications for immuno-oncology are significant.

## Author Contributions

All authors listed have made a substantial, direct, and intellectual contribution to the work and approved it for publication.

## Conflict of Interest Statement

The authors declare that the research was conducted in the absence of any commercial or financial relationships that could be construed as a potential conflict of interest.

## References

[1] Schenkel JM Masopust D. Tissue-resident memory T cells. Immunity (2014) 41(6):886–97. 10.1016/j.immuni.2014.12.007 25526304 PMC4276131

[2] Park CO Kupper TS. The emerging role of resident memory T cells in protective immunity and inflammatory disease. Nat Med (2015) 21(7):688–97. 10.1038/nm.3883 26121195 PMC4640452

[3] Clark RA. Resident memory T cells in human health and disease. Sci Transl Med (2015) 7(269):269rv261. 10.1126/scitranslmed.3010641 25568072 PMC4425129

[4] Mackay LK Braun A Macleod BL Collins N Tebartz C Bedoui S Cutting edge: CD69 interference with sphingosine-1-phosphate receptor function regulates peripheral T cell retention. J Immunol (2015) 194(5):2059–63. 10.4049/jimmunol.1402256 25624457

[5] Watanabe R Gehad A Yang C Scott LL Teague JE Schlapbach C Human skin is protected by four functionally and phenotypically discrete populations of resident and recirculating memory T cells. Sci Transl Med (2015) 7(279):279ra239. 10.1126/scitranslmed.3010302 25787765 PMC4425193

[6] Cerf-Bensussan N Jarry A Brousse N Lisowska-Grospierre B Guy-Grand D Griscelli C A monoclonal antibody (HML-1) defining a novel membrane molecule present on human intestinal lymphocytes. Eur J Immunol (1987) 17(9):1279–85. 10.1002/eji.1830170910 3498635

[7] Kruschwitz M Fritzsche G Schwarting R Micklem K Mason DY Falini B Ber-ACT8: new monoclonal antibody to the mucosa lymphocyte antigen. J Clin Pathol (1991) 44(8):636–45. 10.1136/jcp.44.8.636 1890196 PMC496753

[8] Sheridan BS Lefrancois L. Regional and mucosal memory T cells. Nat Immunol (2011) 12(6):485–91. 10.1038/ni.2029 21739671 PMC3224372

[9] Cepek KL Shaw SK Parker CM Russell GJ Morrow JS Rimm DL Adhesion between epithelial cells and T lymphocytes mediated by E-cadherin and the alpha E beta 7 integrin. Nature (1994) 372(6502):190–3. 10.1038/372190a0 7969453

[10] Schön MP Arya A Murphy EA Adams CM Strauch UG Agace WW Mucosal T lymphocyte numbers are selectively reduced in integrin alpha E (CD103)-deficient mice. J Immunol (1999) 162(11):6641–9. 10352281

[11] Gorfu G Rivera-Nieves J Ley K. Role of beta7 integrins in intestinal lymphocyte homing and retention. Curr Mol Med (2009) 9(7):836–50. 10.2174/156652409789105525 19860663 PMC2770881

[12] Mackay LK Stock AT Ma JZ Jones CM Kent SJ Mueller SN Long-lived epithelial immunity by tissue-resident memory T (TRM) cells in the absence of persisting local antigen presentation. Proc Natl Acad Sci U S A (2012) 109(18):7037–42. 10.1073/pnas.1202288109 22509047 PMC3344960

[13] Casey KA Fraser KA Schenkel JM Moran A Abt MC Beura LK Antigen-independent differentiation and maintenance of effector-like resident memory T cells in tissues. J Immunol (2012) 188(10):4866–75. 10.4049/jimmunol.1200402 22504644 PMC3345065

[14] Jiang X Clark RA Liu L Wagers AJ Fuhlbrigge RC Kupper TS. Skin infection generates non-migratory memory CD8+ T(RM) cells providing global skin immunity. Nature (2012) 483(7388):227–31. 10.1038/nature10851 22388819 PMC3437663

[15] Lycke N. Recent progress in mucosal vaccine development: potential and limitations. Nat Rev Immunol (2012) 12(8):592–605. 10.1038/nri3251 22828912

[16] Wheelock MJ Johnson KR. Cadherin-mediated cellular signaling. Curr Opin Cell Biol (2003) 15(5):509–14. 10.1016/S0955-0674(03)00101-7 14519384

[17] Schlickum S Sennefelder H Friedrich M Harms G Lohse MJ Kilshaw P Integrin alpha E(CD103)beta 7 influences cellular shape and motility in a ligand-dependent fashion. Blood (2008) 112(3):619–25. 10.1182/blood-2008-01-134833 18492951

[18] Le Floc’h A Jalil A Vergnon I Le Maux Chansac B Lazar V Bismuth G Alpha E beta 7 integrin interaction with E-cadherin promotes antitumor CTL activity by triggering lytic granule polarization and exocytosis. J Exp Med (2007) 204(3):559–70. 10.1084/jem.20061524 17325197 PMC2137907

[19] Parker CM Cepek KL Russell GJ Shaw SK Posnett DN Schwarting R A family of beta 7 integrins on human mucosal lymphocytes. Proc Natl Acad Sci U S A (1992) 89(5):1924–8. 10.1073/pnas.89.5.1924 1542691 PMC48566

[20] Hadley GA Bartlett ST Via CS Rostapshova EA Moainie S. The epithelial cell-specific integrin, CD103 (alpha E integrin), defines a novel subset of alloreactive CD8+ CTL. J Immunol (1997) 159(8):3748–56. 9378961

[21] Li MO Flavell RA. TGF-beta: a master of all T cell trades. Cell (2008) 134(3):392–404. 10.1016/j.cell.2008.07.025 18692464 PMC3677783

[22] El-Asady R Yuan R Liu K Wang D Gress RE Lucas PJ TGF-{beta}-dependent CD103 expression by CD8(+) T cells promotes selective destruction of the host intestinal epithelium during graft-versus-host disease. J Exp Med (2005) 201(10):1647–57. 10.1084/jem.20041044 15897278 PMC2212926

[23] Suzuki R Nakao A Kanamaru Y Okumura K Ogawa H Ra C. Localization of intestinal intraepithelial T lymphocytes involves regulation of alphaEbeta7 expression by transforming growth factor-beta. Int Immunol (2002) 14(4):339–45. 10.1093/intimm/14.4.339 11934870

[24] Mackay LK Wynne-Jones E Freestone D Pellicci DG Mielke LA Newman DM T-box transcription factors combine with the cytokines TGF-beta and IL-15 to control tissue-resident memory T cell fate. Immunity (2015) 43(6):1101–11. 10.1016/j.immuni.2015.11.008 26682984

[25] Strutt TM Dhume K Finn CM Hwang JH Castonguay C Swain SL IL-15 supports the generation of protective lung-resident memory CD4 T cells. Mucosal Immunol (2018) 11(3):668–80. 10.1038/mi.2017.101 29186108 PMC5975122

[26] Schenkel JM Fraser KA Casey KA Beura LK Pauken KE Vezys V IL-15-independent maintenance of tissue-resident and boosted effector memory CD8 T cells. J Immunol (2016) 196(9):3920–6. 10.4049/jimmunol.1502337 27001957 PMC5145194

[27] Sathaliyawala T Kubota M Yudanin N Turner D Camp P Thome JJ Distribution and compartmentalization of human circulating and tissue-resident memory T cell subsets. Immunity (2013) 38(1):187–97. 10.1016/j.immuni.2012.09.020 23260195 PMC3557604

[28] Turner DL Farber DL. Mucosal resident memory CD4 T cells in protection and immunopathology. Front Immunol (2014) 5:331. 10.3389/fimmu.2014.00331 25071787 PMC4094908

[29] Hadley GA Charandee C Weir MR Wang D Bartlett ST Drachenberg CB. CD103+ CTL accumulate within the graft epithelium during clinical renal allograft rejection. Transplantation (2001) 72(9):1548–55. 10.1097/00007890-200111150-00013 11707744

[30] Feng Y Wang D Yuan R Parker CM Farber DL Hadley GA. CD103 expression is required for destruction of pancreatic islet allografts by CD8(+) T cells. J Exp Med (2002) 196(7):877–86. 10.1084/jem.20020178 12370250 PMC2194029

[31] Kilshaw PJ Higgins JM Alpha E: no more rejection? J Exp Med (2002) 196(7):873–5. 10.1084/jem.20021404 12370249 PMC2194032

[32] Kuric E Seiron P Krogvold L Edwin B Buanes T Hanssen KF Demonstration of tissue resident memory CD8 T cells in insulitic lesions in adult patients with recent-onset type 1 diabetes. Am J Pathol (2017) 187(3):581–8. 10.1016/j.ajpath.2016.11.002 28212742

[33] Radenkovic M Uvebrant K Skog O Sarmiento L Avartsson J Storm P Characterization of resident lymphocytes in human pancreatic islets. Clin Exp Immunol (2017) 187(3):418–27. 10.1111/cei.12892 27783386 PMC5290249

[34] Barrie ES Lodder M Weinreb PH Buss J Rajab A Adin C Role of ITGAE in the development of autoimmune diabetes in non-obese diabetic mice. J Endocrinol (2015) 224(3):235–43. 10.1530/JOE-14-0396 25525188

[35] Pickup M Novitskiy S Moses HL The roles of TGFbeta in the tumour microenvironment. Nat Rev Cancer (2013) 13(11):788–99. 10.1038/nrc3603 24132110 PMC4025940

[36] Mackay LK Minnich M Kragten NA Liao Y Nota B Seillet C Hobit and Blimp1 instruct a universal transcriptional program of tissue residency in lymphocytes. Science (2016) 352(6284):459–63. 10.1126/science.aad2035 27102484

[37] Milner JJ Toma C Yu B Zhang K Omilusik K Phan AT Runx3 programs CD8(+) T cell residency in non-lymphoid tissues and tumours. Nature (2017) 552(7684):253–7. 10.1038/nature24993 29211713 PMC5747964

[38] Morgan EA Yu H Pinkus JL Pinkus GS. Immunohistochemical detection of hairy cell leukemia in paraffin sections using a highly effective CD103 rabbit monoclonal antibody. Am J Clin Pathol (2013) 139(2):220–30. 10.1309/AJCPHW7RULIZT2GB 23355207

[39] Djenidi F Adam J Goubar A Durgeau A Meurice G de Montpréville V CD8+CD103+ tumor-infiltrating lymphocytes are tumor-specific tissue-resident memory T cells and a prognostic factor for survival in lung cancer patients. J Immunol (2015) 194(7):3475–86. 10.4049/jimmunol.1402711 25725111

[40] Wang ZQ Milne K Derocher H Webb JR Nelson BH Watson PH. CD103 and intratumoral immune response in breast cancer. Clin Cancer Res (2016) 22(24):6290–7. 10.1158/1078-0432.CCR-16-0732 27267849

[41] Webb JR Milne K Nelson BH. PD-1 and CD103 are widely coexpressed on prognostically favorable intraepithelial CD8 T cells in human ovarian cancer. Cancer Immunol Res (2015) 3(8):926–35. 10.1158/2326-6066.CIR-14-0239 25957117

[42] Workel HH Komdeur FL Wouters MC Plat A Klip HG Eggink FA CD103 defines intraepithelial CD8+ PD1+ tumour-infiltrating lymphocytes of prognostic significance in endometrial adenocarcinoma. Eur J Cancer (2016) 60:1–11. 10.1016/j.ejca.2016.02.026 27038842

[43] Komdeur FL Wouters MC Workel HH Tijans AM Terwindt AL Brunekreeft KL CD103+ intraepithelial T cells in high-grade serous ovarian cancer are phenotypically diverse TCRalphabeta+ CD8alphabeta+ T cells that can be targeted for cancer immunotherapy. Oncotarget (2016) 7(46):75130–44. 10.18632/oncotarget.12077 27650547 PMC5342728

[44] Komdeur FL Prins TM van de Wall S Plat A Wisman GBA Hollema H CD103+ tumor-infiltrating lymphocytes are tumor-reactive intraepithelial CD8+ T cells associated with prognostic benefit and therapy response in cervical cancer. Oncoimmunology (2017) 6(9):e1338230. 10.1080/2162402X.2017.1338230 28932636 PMC5599086

[45] Edwards J Wilmott JS Madore J Gide TN Quek C Tasker A CD103(+) tumor-resident CD8(+) T cells are associated with improved survival in immunotherapy-naive melanoma patients and expand significantly during anti-PD-1 treatment. Clin Cancer Res (2018) 24(13):3036–45. 10.1158/1078-0432.CCR-17-2257 29599411

[46] Ganesan AP Clarke J Wood O Garrido-Martin EM Chee SJ Mellows T Tissue-resident memory features are linked to the magnitude of cytotoxic T cell responses in human lung cancer. Nat Immunol (2017) 18(8):940–50. 10.1038/ni.3775 28628092 PMC6036910

[47] Quinn E Hawkins N Yip YL Suter C Ward R CD103+ intraepithelial lymphocytes – a unique population in microsatellite unstable sporadic colorectal cancer. Eur J Cancer (2003) 39(4):469–75. 10.1016/S0959-8049(02)00633-0 12751377

[48] Boddupalli CS Bar N Kadaveru K Krauthammer M Pornputtapong N Mai Z Interlesional diversity of T cell receptors in melanoma with immune checkpoints enriched in tissue-resident memory T cells. JCI Insight (2016) 1(21):e88955. 10.1172/jci.insight.88955 28018970 PMC5161225

[49] Ling KL Dulphy N Bahl P Salio M Maskell K Piris J Modulation of CD103 expression on human colon carcinoma-specific CTL. J Immunol (2007) 178(5):2908–15. 10.4049/jimmunol.178.5.2908 17312135

[50] Cresswell J Robertson H Neal DE Griffiths TR Kirby JA. Distribution of lymphocytes of the alpha(E)beta(7) phenotype and E-cadherin in normal human urothelium and bladder carcinomas. Clin Exp Immunol (2001) 126(3):397–402. 10.1046/j.1365-2249.2001.01652.x 11737053 PMC1906227

[51] Lohneis P Sinn M Bischoff S Jühling A Pelzer U Wislocka L Cytotoxic tumour-infiltrating T lymphocytes influence outcome in resected pancreatic ductal adenocarcinoma. Eur J Cancer (2017) 83:290–301. 10.1016/j.ejca.2017.06.016 28772128

[52] Guo X Zhang Y Zheng L Zheng C Song J Zhang Q Global characterization of T cells in non-small-cell lung cancer by single-cell sequencing. Nat Med (2018) 24(7):978–85. 10.1038/s41591-018-0045-3 29942094

[53] Savas P Virassamy B Ye C Salim A Mintoff CP Caramia F Single-cell profiling of breast cancer T cells reveals a tissue-resident memory subset associated with improved prognosis. Nat Med (2018) 24(7):986–93. 10.1038/s41591-018-0078-7 29942092

[54] Webb JR Wick DA Nielsen JS Tran E Milne K McMurtrie E Profound elevation of CD8+ T cells expressing the intraepithelial lymphocyte marker CD103 (alphaE/beta7 Integrin) in high-grade serous ovarian cancer. Gynecol Oncol (2010) 118(3):228–36. 10.1016/j.ygyno.2010.05.016 20541243

[55] Webb JR Milne K Watson P Deleeuw RJ Nelson BH. Tumor-infiltrating lymphocytes expressing the tissue resident memory marker CD103 are associated with increased survival in high-grade serous ovarian cancer. Clin Cancer Res (2014) 20(2):434–44. 10.1158/1078-0432.CCR-13-1877 24190978

[56] Webb JR Milne K Nelson BH. Location, location, location: CD103 demarcates intraepithelial, prognostically favorable CD8+ tumor-infiltrating lymphocytes in ovarian cancer. Oncoimmunology (2014) 3(1):e27668. 10.4161/onci.27668 25101220 PMC4121334

[57] Bösmüller HC Wagner P Peper JK Schuster H Pham DL Greif K Combined immunoscore of CD103 and CD3 identifies long-term survivors in high-grade serous ovarian cancer. Int J Gynecol Cancer (2016) 26(4):671–9. 10.1097/IGC.0000000000000672 26905331

[58] Santoiemma PP Reyes C Wang LP McLane MW Feldman MD Tanyi JL Systematic evaluation of multiple immune markers reveals prognostic factors in ovarian cancer. Gynecol Oncol (2016) 143(1):120–7. 10.1016/j.ygyno.2016.07.105 27470997

[59] Milne K Barnes RO Girardin A Mawer MA Nesslinger NJ Ng A Tumor-infiltrating T cells correlate with NY-ESO-1-specific autoantibodies in ovarian cancer. PLoS One (2008) 3(10):e3409. 10.1371/journal.pone.0003409 18923710 PMC2561074

[60] Malik BT Byrne KT Vella JL Zhang P Shabaneh TB Steinberg SM Resident memory T cells in the skin mediate durable immunity to melanoma. Sci Immunol (2017) 2(10). 10.1126/sciimmunol.aam6346 28738020 PMC5525335

[61] Boniface K Jacquemin C Darrigade AS Dessarthe B Martins C Boukhedouni N Vitiligo skin is imprinted with resident memory CD8 T cells expressing CXCR3. J Invest Dermatol (2018) 138(2):355–64. 10.1016/j.jid.2017.08.038 28927891

[62] Simoni Y Becht E Fehlings M Loh CY Koo SL Teng KWW Bystander CD8(+) T cells are abundant and phenotypically distinct in human tumour infiltrates. Nature (2018) 557(7706):575–9. 10.1038/s41586-018-0130-2 29769722

[63] Posavad CM Zhao L Dong L Jin L Stevens CE Magaret AS Enrichment of herpes simplex virus type 2 (HSV-2) reactive mucosal T cells in the human female genital tract. Mucosal Immunol (2017) 10(5):1259–69. 10.1038/mi.2016.118 28051084 PMC5496807

[64] Rodriguez-Garcia M Fortier JM Barr FD Wira CR Aging impacts CD103(+) CD8(+) T cell presence and induction by dendritic cells in the genital tract. Aging Cell (2018) 17(3):e12733 10.1111/acel.12733 29455474 PMC5946085

[65] Lavoué V Thédrez A Levêque J Foucher F Henno S Jauffret V Immunity of human epithelial ovarian carcinoma: the paradigm of immune suppression in cancer. J Transl Med (2013) 11:147. 10.1186/1479-5876-11-147 23763830 PMC3683338

[66] Melichar B Nash MA Lenzi R Platsoucas CD Freedman RS. Expression of costimulatory molecules CD80 and CD86 and their receptors CD28, CTLA-4 on malignant ascites CD3+ tumour-infiltrating lymphocytes (TIL) from patients with ovarian and other types of peritoneal carcinomatosis. Clin Exp Immunol (2000) 119(1):19–27. 10.1046/j.1365-2249.2000.01105.x 10606960 PMC1905534

[67] Curiel TJ Coukos G Zou L Alvarez X Cheng P Mottram P Specific recruitment of regulatory T cells in ovarian carcinoma fosters immune privilege and predicts reduced survival. Nat Med (2004) 10(9):942–9. 10.1038/nm1093 15322536

[68] Kryczek I Zou L Rodriguez P Zhu G Wei S Mottram P B7-H4 expression identifies a novel suppressive macrophage population in human ovarian carcinoma. J Exp Med (2006) 203(4):871–81. 10.1084/jem.20050930 16606666 PMC2118300

[69] Obermajer N Muthuswamy R Odunsi K Edwards RP Kalinski P. PGE(2)-induced CXCL12 production and CXCR4 expression controls the accumulation of human MDSCs in ovarian cancer environment. Cancer Res (2011) 71(24):7463–70. 10.1158/0008-5472.CAN-11-2449 22025564 PMC4993027

[70] Zhu X Lang J. The significance and therapeutic potential of PD-1 and its ligands in ovarian cancer: a systematic review. Gynecol Oncol (2016) 142(1):184–9. 10.1016/j.ygyno.2016.04.002 27063803

[71] Hombrink P Helbig C Backer RA Piet B Oja AE Stark R Programs for the persistence, vigilance and control of human CD8(+) lung-resident memory T cells. Nat Immunol (2016) 17(12):1467–78. 10.1038/ni.3589 27776108

[72] Amsen D Hombrink P van Lier RAW Tumor immunity requires border patrol to fight the enemy within. Nat Immunol (2017) 18(8):870–2. 10.1038/ni.3792 28722713

